# Design of a 3D patient‐specific collision avoidance virtual framework for half‐gantry proton therapy system

**DOI:** 10.1002/acm2.13496

**Published:** 2021-12-10

**Authors:** Jingjing M. Dougherty, Thomas J. Whitaker, Daniel W. Mundy, Erik J. Tryggestad, Chris J. Beltran

**Affiliations:** ^1^ Department of Radiation Oncology Mayo Clinic Jacksonville Florida USA; ^2^ Department of Radiation Physics MD Anderson Cancer Center Houston Texas USA; ^3^ Department of Radiation Oncology Mayo Clinic Rochester Minnesota USA

**Keywords:** API scripting, CAD model, collision avoidance, ESAPI, proton beam configurations, proton therapy

## Abstract

**Introduction:**

This study presents a comprehensive collision avoidance framework based on three‐dimension (3D) computer‐aided design (CAD) modeling, a graphical user interface (GUI) as peripheral to the radiation treatment planning (RTP) environment, and patient‐specific plan parameters for intensity‐modulated proton therapy (IMPT).

**Methods:**

A stand‐alone software application was developed leveraging the Varian scripting application programming interface (API) for RTP database object accessibility. The Collision Avoider software models the Hitachi ProBeat‐V half gantry design and the Kuka robotic couch with triangle mesh structures. Patient‐specific plan parameters are displayed in the collision avoidance software for potential proximity evaluation. The external surfaces of the patients and the immobilization devices are contoured based on computed tomography (CT) images. A “table junction‐to‐CT‐origin” (JCT) measurement is made for every patient at the time of CT simulation to accurately provide reference location of the patient contours to the treatment couch. Collision evaluations were performed virtually with the program during treatment planning to prevent four major types of collisional events: collisions between the gantry head and the treatment couch, gantry head and the patient's body, gantry head and the robotic arm, and collisions between the gantry head and the immobilization devices.

**Results:**

The Collision Avoider software was able to accurately model the proton treatment delivery system and the robotic couch position. Commonly employed clinical beam configuration and JCT values were investigated. Brain and head and neck patients require more complex gantry and patient positioning system configurations. Physical measurements were performed to validate 3D CAD model geometry. Twelve clinical proton treatment plans were used to validate the accuracy of the software. The software can predict all four types of collisional events in our clinic since its full implementation in 2020.

**Conclusion:**

A highly efficient patient‐specific collision prevention program for scanning proton therapy has been successfully implemented. The graphical program has provided accurate collision detection since its inception at our institution.

## 1 INTRODUCTION

Intensity‐modulated proton therapy (IMPT) often requires complex beam configurations for robust tumor target irradiation. Collision events during treatment sessions (even those discovered before any actual interference occurs) negatively impact the quality of patients’ treatment experience and pose a potentially significant safety concern. The primary solution after a collision event occurs is to immediately replan with a set of new validated gantry or couch angles for the remaining fractions. An unforeseen replanning event adds inefficiencies or workflow perturbations to a busy proton clinic and, more importantly, has the potential to impact patients’ prognoses. For example, for single‐fraction stereotactic radiosurgery or hypofractionated stereotactic body radiotherapy (RT) proton treatments, a collision event could further complicate the biological effectiveness, if one or more of the treatment fields must be replanned and delivered later. Or, patients’ RT treatment start date or treatment fractionation schedule may be impacted. The purpose of this study is to present a clinically validated collision avoidance framework that has been implemented at our clinic.

Currently, there are two categories of collision detection solutions available in the field of radiation therapy. First, vendor provided software modules, either based on a simple treatment machine geometry modeling or a more sophisticated three‐dimension (3D) computer‐aided design (CAD) modeling. The second class of solutions is a group of user‐initiated, practical in‐house techniques including: mathematical approximation of collision‐free space,^1^, ^2^, ^3^, ^4^, ^5^ stand‐alone graphical simulation software,^6^, ^7^, ^8^, ^9^, ^10^ using scripting application programming interface (API) to build integrated 3D collision detection software,^11^, ^12^, ^13^ and using a priori patient and machine surface model to predict collision‐free space.^14^, ^15^, ^16^, ^17^, ^18^ Both the vendor licensed and the home‐built collision detection methods have been widely investigated and adapted for photon LINAC‐based clinical workflow. Practical solutions for proton therapy system have not been adequately reported. Unlike the LINAC designs by two major vendors, proton therapy system designs differ dramatically across vendors and even within a single vendor itself. This machine geometry variation makes it challenging to provide one‐size fits all collision detection solutions to all or majority of the proton therapy centers.

In 1995, Kessler et al. published the first interactive CAD‐based simulation method to account for collision issues between a LINAC gantry and its treatment couch.^19^ Zou et al. later extended the method by incorporating 3D patient‐specific geometry and a ray‐tracing algorithm to automatically detect collisional events between a proton gantry and patients.^8^ However, the proton system they modeled was based on a 360‐degree full gantry design, which has different collision concerns when compared to a 180‐degree half gantry system. Mainly, the impact between the nozzle and the treatment couch or the robotic support arm can be challenging for the half gantry system. Conversely, a full gantry system may easily circumvent the potential issues by simply changing the gantry or couch angles. To our best knowledge, there has not been any publication comprehensively to address various types of collisions for a half gantry proton system.

Categorically, there are mainly four types of collision scenarios during a proton therapy treatment delivery for a half gantry proton system: (1) collisions between the gantry nozzle and the couch top, (2) collisions between the gantry nozzle and the patient, (3) collisions between the gantry nozzle with the robotic patient positioning system (PPS), and (4) collisions between the gantry and the patient immobilization devices. For shallow treatment volumes, a range shifter device needs to be manually attached to the downstream or exit side of the proton nozzle, compounding collision hazards. With the four collisional settings in mind, we aim to present a comprehensive collision avoidance framework that has been fully integrated with our clinical workflow. We created a stand‐alone Windows application known as “Collision Avoider” using the Eclipse Scripting API (ESAPI, Varian Medical Systems Inc.) and Microsoft Visual Studio (MVS, Microsoft, Inc.).

## 2 METHODS

### 2.1 CAD modeling

**FIGURE 1:**
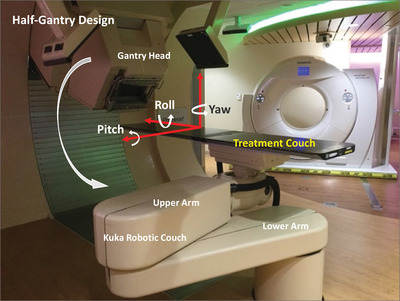
One of the four identical treatment room configurations for the Hitachi ProBeat‐V proton therapy system. The gantry rotates from 355° to 185°. The Kuka robotic couch has a left elbow configuration capable of six degrees of freedom positional corrections

**FIGURE 2:**
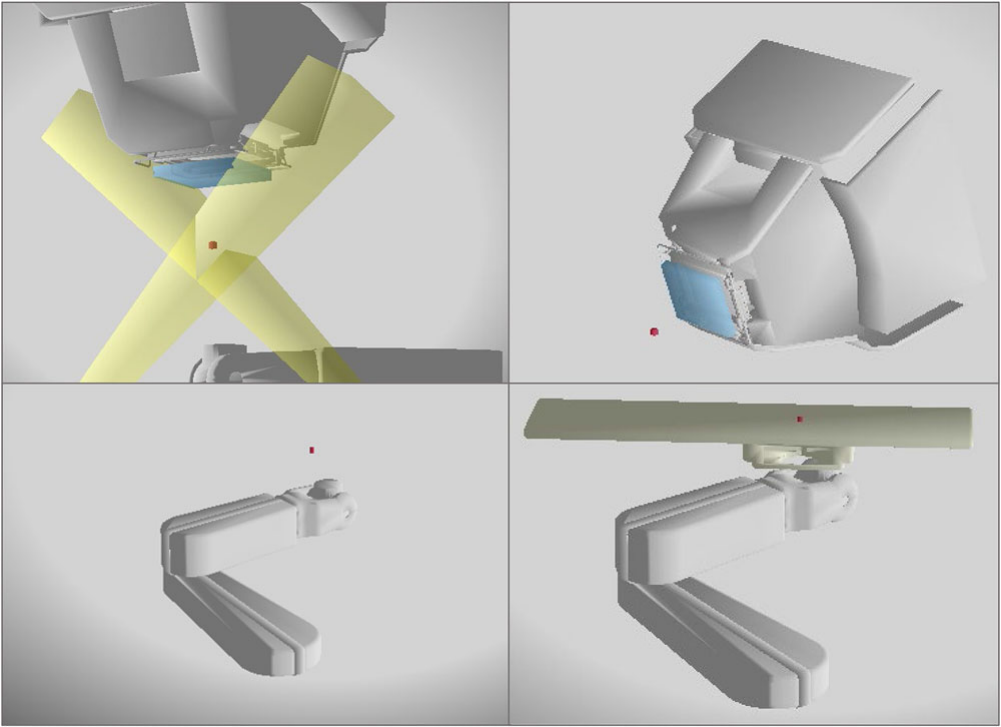
Three‐dimensional computer aided design (CAD) models for the Hitachi ProBeat‐V gantry head and the Kuka robotic arm with CIVCO couch top

The Collision Avoider contains a detailed 3D CAD model of the Hitachi ProBeat‐V proton gantry head and PPS provided by the manufacturer (Hitachi Americas, Ltd.), which is currently installed at two campuses, namely Mayo Clinic Rochester, Minnesota, and Mayo Clinic Phoenix, Arizona. We defined the gantry and PPS to be moved according to the International Electrotechnical Commission convention. The Hitachi gantry has a half gantry design capable of rotating from 355° to 185° (Figure 1). The Gantry CAD model provided relevant details of the gantry body, head casing and the touch guard system (Figure 2). The extended range shifter device was modeled as a separate insertable 3D CAD component. The physical dimension of all input CAD models has been validated through independent measurements. The distance between the proton exit window (on the gantry nozzle) and the treatment room isocenter has also been validated both in the CAD model and in the treatment rooms. In addition, the distance between the external range shifter and the room isocenter has also been experimentally measured. Our centers employ a PPS that has a left elbow configuration or “left‐handedness.” The PPS itself contains three separate CAD components: the base and the lower arm, the upper arm and the knuckle, and the treatment couch. Coupled to the PPS necessarily is an interchangeable couch support depending on treatment site (Figure 2). The two general types of couch supports routinely utilized clinically at Mayo Clinic Rochester are the CIVCO Universal couch top (CIVCO Radiotherapy, Iowa, USA) and the Orfit HP Pro extension (Orfit, Wijnegem, Belgium). In addition to the physical hardware components, we also generated a model for the in‐room oblique stereoscopic kV X‐Ray fields used for image guidance to help visualize the potential obstruction of kV radiographs by the gantry head or PPS during treatment.

### 2.2 ESAPI stand‐alone application

The Collision Avoider software was created using C#/.NET in the MVS development environment (Microsoft, Inc.), incorporating published libraries from the Varian ESAPI. The Collision Avoider software package was created as a stand‐alone executable .NET application. The advantage of a stand‐alone executable deployment scheme is mainly efficiency: while the ESAPI plug‐in scripts (which are necessarily deployed inside the Eclipse GUI) are confined to one active loaded patient file at a time, the stand‐alone executables can gain access to the entire Eclipse radiation treatment planning (RTP) patient database and access any ESAPI‐handled RTP objects from any existing patient. Users can run multiple collision checks in series without interfering with their planning progress in the Eclipse treatment planning system.

**FIGURE 3:**
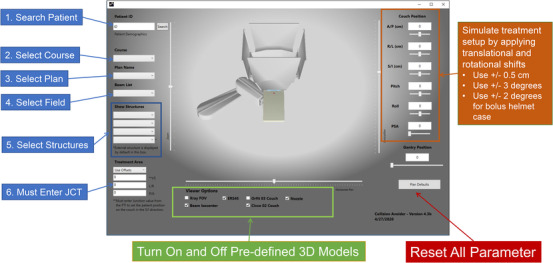
Graphical user interface (GUI) design of the Collision Avoider software

The graphical user interface (GUI) of the Collision Avoider is shown in Figure 3. On the left‐hand side of the software display, the user can search and select patient plan information. The bottom of the window allows users to select plan‐specific 3D CAD models. For instance, if the extended range shifter is used in the plan, the collision avoidance needs to be visually inspected by selecting the "ERS45" device. On the right‐hand side, the user can simulate a patient setup by applying additional rotation and translational corrections. The center of the software window displays the patient‐specific contours and the position of the 3D CAD modeling of the proton system according to a plan. The user can freely pan and zoom the model display by changing its setting with a slider in the 3D model space.

**FIGURE 4:**
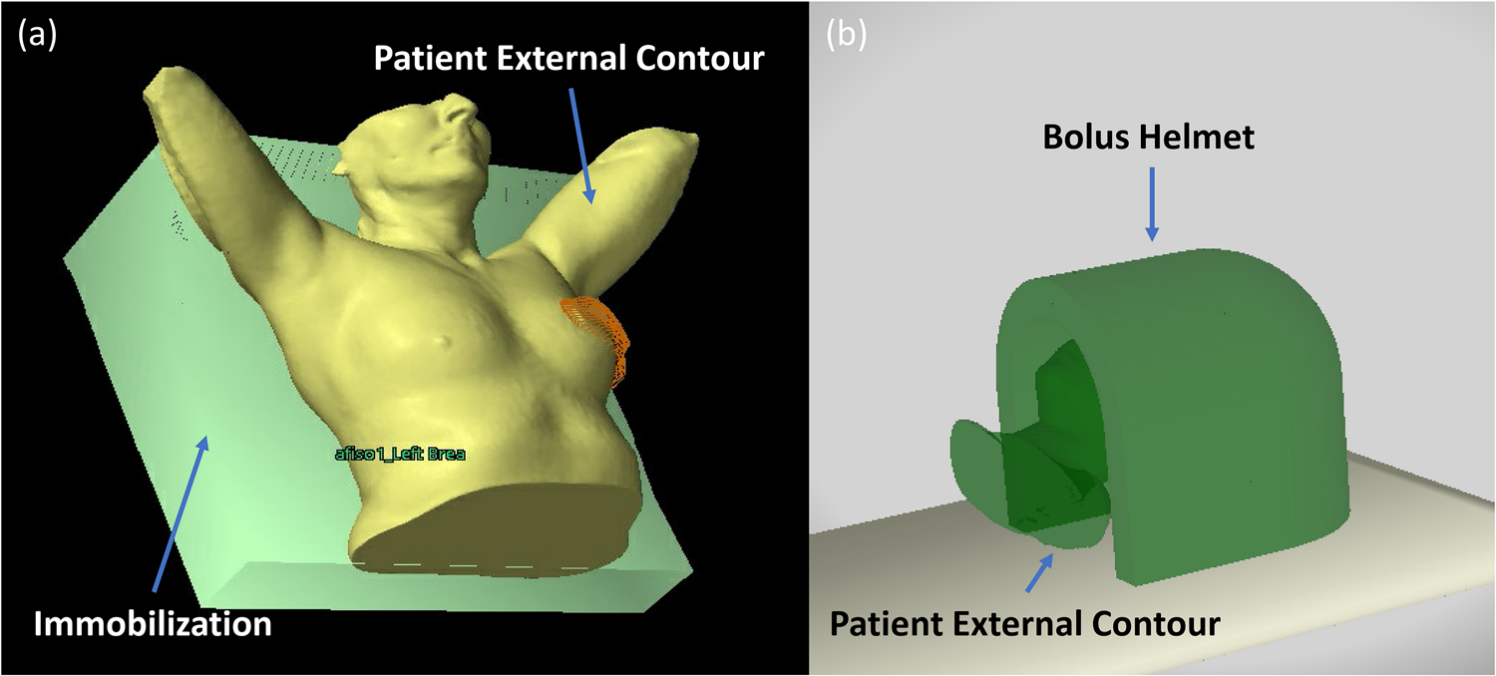
(a) A breast patient external geometry from computed tomography (CT) image contours and coarse contour of the breast board immobilization device. (b) A patient external contour and bolus helmet structure from CT image contours

The patient‐specific RTP objects or parameters are queried automatically through the Eclipse patient database using the patient ID (or medical record number). The software user then identifies the proton plan by selecting the corresponding course name and plan name. A list of the beams is then populated automatically according to the selected proton plan. The 3D graphical display will then automatically rotate the gantry and the robotic couch according to the selected beam. The Collision Avoider retrieves all patient‐derived structures (i.e., regions‐of‐interest) from the Aria database (associated with the selected plan) and displays up to four structures at a time. The RT structures were converted into 3D mesh objects to support their 3D representation within the GUI. All structures are automatically positioned to the corresponding planned beam isocenter. The most useful patient structure to evaluate potential interference between the proton gantry head and the patient is the external or body contour (outer skin boundary). Our clinical practice also includes dosimetrists contouring patient‐specific rigid immobilization as well as tertiary range shifter (or range pullback) devices, including but not limited to a custom Vac‐Lok (CIVCO) or Klarity Cushion (Klarity Medical, Ohio, USA) supporting the patient, foam padding for patient comfort, in‐house designed and fabricated bolus helmets,^20^ and the breast board base (Figure 4).

### 2.3 Clinical workflow

**FIGURE 5:**
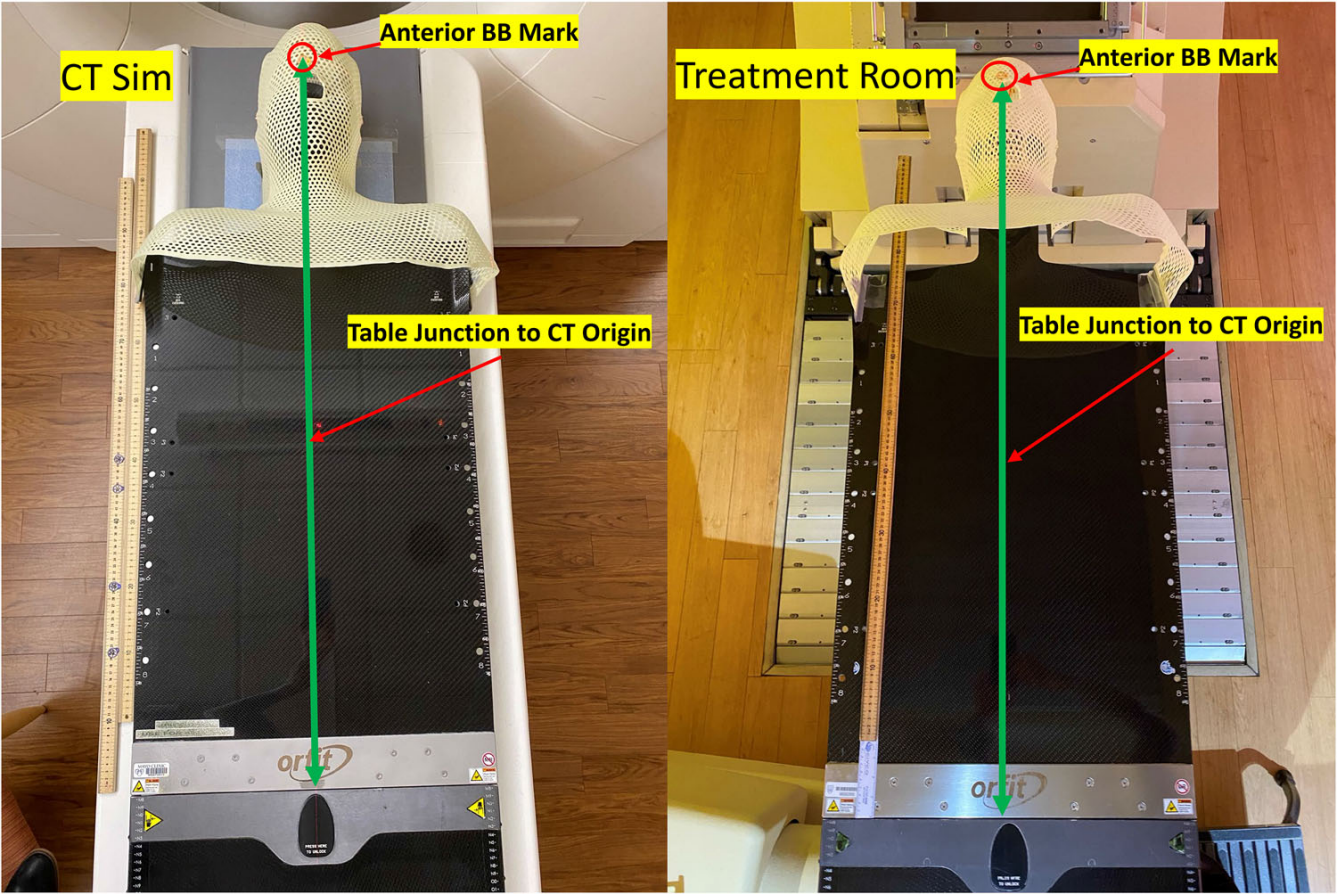
Definition of the “table junction to computed tomography (CT) origin” or junction measurements in CT simulation room and treatment room. The green line with double arrows represents the junction length for a head and neck patient with five‐point thermoplastic mask on an Orfit couch support.

As part of standard clinical workflow, a “table junction‐to‐computed tomography (CT) origin” (i.e., “junction”) measurement is made at the time of CT simulation for every proton patient by the therapists and entered manually into the electronic medical record. This junction value is defined as the distance between the couch extension connection junction and the CT simulation reference or isocenter mark (Figure 5). The CT simulation reference mark is subsequently set as the CT coordinate origin in Eclipse during RTP. Thus, the junction parameter provides the necessary indexing for the display of patient‐derived structures on the 3D CAD treatment couch within Collision Avoider. We extracted the JCT values from 140 patients’ ARIA (an information software enviroment for radiation, medical, and surgical oncology) documentation to investigate its mean and standard deviation for each treatment disease site.

**FIGURE 6:**
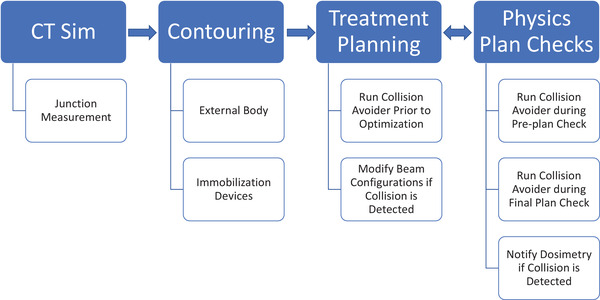
Clinical workflow of the virtual collision detection process is presented here

The dosimetrist will create an external body or boundary contour based on the CT simulation images. This body structure is logically the primary 3D geometry used for collision evaluation with the Hitachi gantry head. Any immobilization devices that extend outside of the treatment couch will need to be contoured in the treatment planning system. Based on the specific clinical workflow employed at Mayo Clinic Rochester, once the dosimetrist completes the contouring process and picks the appropriate beam angles, the preliminary treatment plan will be sent for a “pre‐plan check” by a medical physicist. As part of this task, the Collision Avoider software is run by both the dosimetrist and the medical physicist to graphically verify the beam angles, which are achievable before the optimization process starts in Eclipse. Once the plan has been approved by the physician, clinical workflow stipulates that a secondary medical physicist performs the final plan check which includes re‐running the Collision Avoider software to confirm that the beams are deliverable prior to the first fraction patient treatment. During either the pre‐plan check or the final plan check, if a beam fails the collision evaluation, a new beam angle is selected, and a new plan will be created. The clinical collision detection process has been summarized in Figure 6.

As additional safety precaution, we employ a 3‐cm buffer margin such that when the distance between the 3D CAD models or patient contours becomes less than 3 cm, the user should send the beam configuration in question for a physical "angle check" on the treatment machine at the end of the day. The therapists generally perform this task independently (with a medical physicist available for support when needed) using the junction reference length and the beam parameters from plan. The supplied junction parameter provides an indexing reference mark where the patient would be on the treatment couch top. If additional three‐point shifts are required (to the isocenter location), the therapists will shift the PPS coordinate according to the treatment plan. In most angle‐check scenarios, patient immobilization devices such as the Orfit thermoplastic mask, breast board, or the Vac‐Lok are placed on the couch to evaluate potential impacts.

### 2.4 Beam configurations

To define the beam angle complexity for each disease site, we investigated the commonly employed RTP couch and gantry angles, using spreadsheet records manually maintained by our dosimetrists and RTP Aria database queries from prior publication.^21^

## 3 RESULTS

### 3.1 Validation studies

The validation of the Collision Avoider software consists of both 3D CAD model validation and evaluation with clinical treatment plans. Rigorous measurements were performed in the gantry rooms to validate the 3D CAD models. The following geometric validation was performed with gantry head and touch guard dimension, ERS45 device dimension, dimensions of the couch tops, dimensions of the robotic arms, distance between the laser isocenter and the base of the robotic arms, distance between the laser isocenter and the ERS45 device, and distance between the laser isocenter and the face of the gantry nozzle. Based on the validation, we estimated that uncertainty associated with the CAD models are less than 1 cm. The biggest uncertainty comes from the couch top modeling, since additional 1‐cm margin was built into the model as an additional safety precaution in the longitudinal direction. The initial testing of the Collision Avoider was performed using clinical treatment plans from 12 patients (one CSI, two brain, three breast, three GI/GU, and three head and neck), four of which with known true positive colliding beam configurations (one breast, one GI, one brain, and one head and neck). The Collision Avoider was able to predict all four true positive colliding beam configurations and eight true negative cases. No false positive configurations were reported. The four major types of collisional scenarios are reported in Table 1, along with corresponding disease sites where impacts are most likely to occur based on clinical observations. For head and neck cases, the proximity issue generally occurs on the patient's shoulder region and on the Orfit couch top where the patient's shoulders are located. Since a range shifter is attached on the gantry nozzle for these types of treatments, the range shifter is the primary concern when evaluating potential collisions. The primary proximity concern for a breast or a chestwall patient is the ERS45 device colliding with the patient's arm. Even though our clinical practice is minimizing the arm angle with respect to the couch as much as possible, some patients require a higher arm position due to surgical pain, at the risk of collisions with the ERS45 device or the gantry head touch guard. The standard clinical beam orientations for esophageal patients are two to three posterior or posterior oblique beam angles. A range shifter device is generally not needed for treatment planning and delivery. The primary proximity concern for esophagus treatment is the collision of the gantry head cover with the upper robotic arm.

**TABLE 1:** The four major types of collision scenarios found in different treatment disease sites

Collisional scenarios	Disease sites
Gantry head colliding with the treatment couch top	Head and neck, brain, GI/ GU, breast
Gantry head colliding with the robotic arm	GI/GU
Gantry head colliding with the patient	Breast, head, and neck
Gantry head colliding with the immobilization device	Breast

Abbreviations: GI, gastrointestinal; GU, genitourinary.

### 3.2 Beam configurations

**FIGURE 7:**
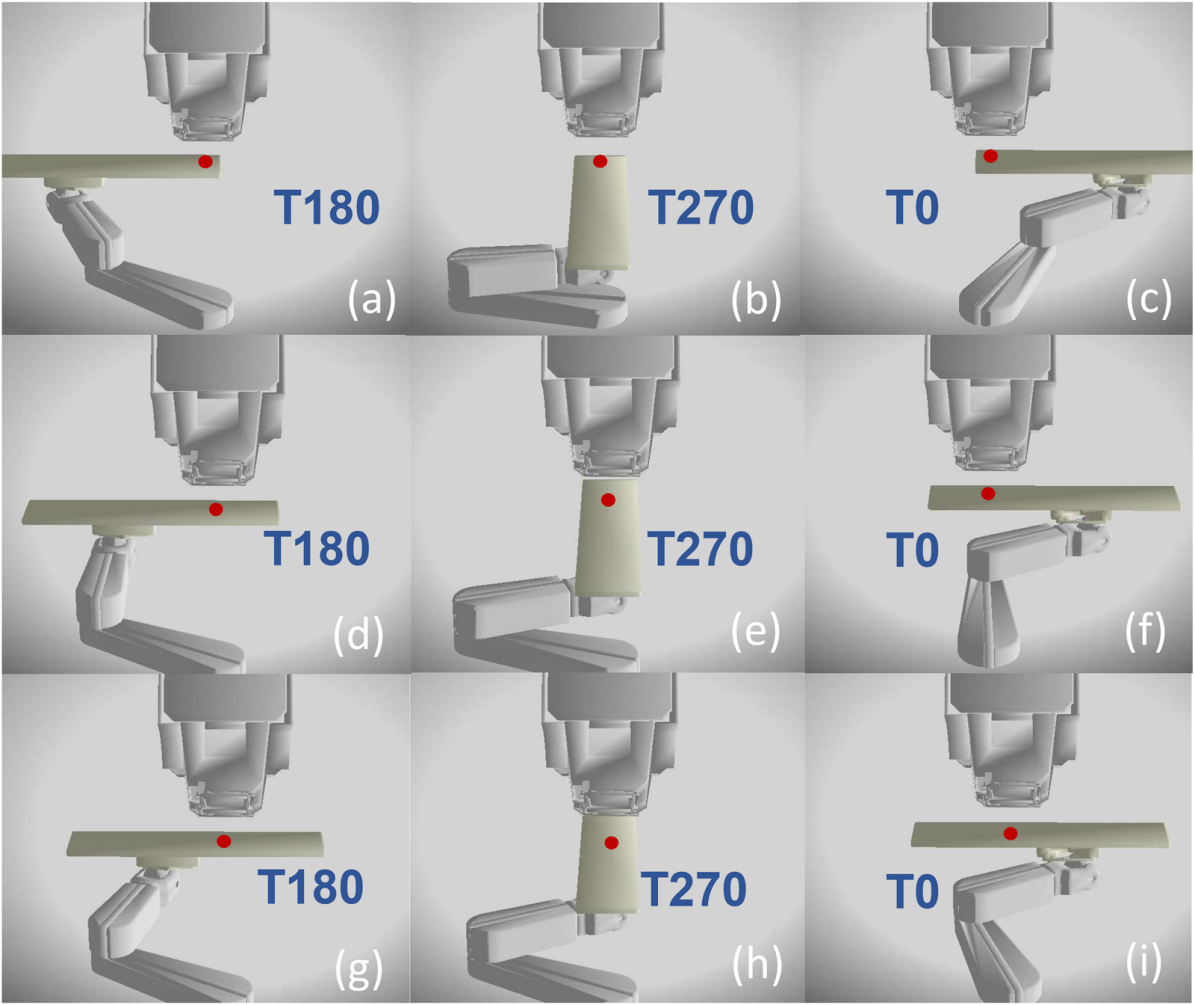
Robotic arm and couch positions for various treatment isocenters. (a‐c) Treatment isocenter located at brain. (d‐f) Treatment isocenter located at the thorax. (g‐i) Treatment isocenter located near the pelvis. As a reference, the gantry rotates in the plane parallel to the table long axis at T270. The red dot represents the radiation isocenter. The gantry head is at 45‐degree rotation

Table 2 lists the most employed gantry and couch configurations for each of the disease sites at Mayo Clinic Rochester. The T0 and T180 couch angles are the most frequently used across all disease sites. The proton plans for the brain disease site utilize the largest range of gantry angles as well as the largest table angles for noncoplanar beam configurations. Among various brain treatments, cases with bolus helmets have the most potential of a collisional event between the top corners of the helmet baseplate and the gantry head. The head and neck treatments employ the next most complex beam configurations with the collisional possibility occurring between the shoulder regions of the patient or tabletop with the gantry head. The mean and the standard deviation of the junction values, distance between the CT simulation isocenter (BB marks) and the CT/treatment couch junction, are presented in Table 2. The junction values vary across different treatment disease sites. The values are smaller for inferior treatment locations and higher when the reference marks are set superiorly to the patient's body. For smaller junction values, like the treatments around the pelvis region, the posterior or posterior oblique beam angles have a higher collisional potential between the gantry head and the robotic arm (shown in Figure 7). For large junctional values greater than 90 cm, like the treatments around the brain or the head and neck regions, the lateral or oblique beam configurations have a higher collisional potential between the gantry head and corners of the couch tops, as well as between the ERS45 range shifter and the patient's shoulders.

**TABLE 2:** Common beam configurations and junction values for various disease sites

Disease site	JCT values (cm, *n* = 20 per site)	Couch angle	Gantry angle
Head and neck	94.7 ± 5.6	T = 180°–195° T = 270° T = 0°	G = 10°–35° G = 40°–50° G = 80°–110° G = 150°–160° G = 175°–180°
GI	67 ± 5.8	T = 350°–0° T = 175°–180°	G = 150°–165°
GU	53.5 ± 6.7	T = 0° T = 180°	G = 50° G = 90°
Breast/Chestwall	54.2 ± 2.6	T = 0° T = 180°	G = 0° G = 15°–55° G = 110°
CSI	111 ± 5.7	T = 0° T = 180° T = 270°	G = 180° G = 45°–90° G = 120°–135°
Brain	105.7 ± 3.2	T = 0°–350° T = 250°–270° T = 180°–200°	G = 30°–80° G = 90° G = 120°–160° G = 180°
Thorax	75.4 ± 10.3	T = 350°–0° T = 180°	G = 0° G = 30°–45° G = 90° G = 145°–160° G = 180°

Abbreviations: CSI, craniospinal irradiation; GI, gastrointestinal; GU, genitourinary; JCT, junction‐to‐computed tomography (CT)‐origin.

### 3.3 Summary of clinical operation with collision avoider

With the official updated clinical release of the Collision Avoider software between May of 2020 and June of 2021, a total of 1418 unique treatment courses have occurred at our facility. No on‐treatment collision events have been reported due to the inaccuracy of the Collision Avoider software. Our virtual collision check process with the software was able to correctly predict and prevent collisional events. Based on anecdotal experience, the time it requires to run a beam angle check with the Collision Avoider software is typically less than 10 min. The total time it takes to perform an actual angle check on the machine is 10–20 min depending on plan complexity.

## 4 DISCUSSION

We have successfully implemented a virtual collision detection process at our proton center to increase clinical efficiency and improve patient treatment safety. The reported methods can prevent problematic beam configurations with respect to the four common types of collisional scenarios encounter in our proton therapy clinic. Our collision avoidance workflow is quite thoroughly integrated with our current clinical workflow. Similar CAD modeling‐based proton studies have been reported before,^8^, ^14^ but we believe the scope of Collision Avoider capabilities are novel in that this tool can address all four types of collision issues as presented in this study.

Although it is relatively uncommon that immobilization devices protrude outside of the treatment couch top boundary, collision incidents are more likely to occur if these devices are not considered in the Collision Avoider software. Our clinical practice requires these Vac‐Lok devices to be included in the CT scan and contoured by our dosimetrists if they are adjacent to the treatment target. Such practice requires little effort up front but provides important benefits later in collision prevention.

Being able to confidently predict where potential collision might occur can potentially increase a planner's creativity in selecting better beam configurations. Of all the treatment disease sites, IMPT treatment planning for the head and neck and brain targets require the most complex noncoplanar beam configurations. Our Collision Avoider GUI provides additional interactive methods for planners to select potential feasible gantry and couch angles based on a patient's external body contour and immobilization devices. We have estimated the number of modifications to the beam configuration is disease site dependent. The percent modification is the highest for the head and neck disease site with 30% of the beam configuration needed modification during the planning process based on the feedback from the Collision Avoider.

We should mention that the deployment of the Collision Avoider software has been implemented in two separate phases. The initial beta testing version released prior to 2020 has been extremely important in eliciting user feedback from all workgroups in our department. The validated clinical version released in May 2020 was enhanced based on the feedback collected. The Collision Avoider software currently only provides graphical validations through user input selections; a simple "pass" or "fail" output can be generated to decrease user interaction for each beam configuration. In addition, it may be beneficial to add a separate module to automatically detect low density immobilization devices in the CT image set to alert users.

Furthermore, original inception of Collision Avoider relied on a local install of Aria (Varian Medical Systems, Inc.) software; more recently, Collision Avoider was deployed as a stand‐alone Citrix application to all radiation oncology stakeholders and is now being maintained by information technology staff, which improves accessibility and scalability within our busy clinical environment.

In a broader context, the future fully or semi‐automated beam angle optimization (BAO) for a half gantry proton system relies on a highly accurate and efficient collision avoidance solution. A rich database of prior workable beam configurations can be established for complex disease sites per clinic‐specific practice. Synchronization between the mathematical solutions to BAO problem and a practical beam configuration database can potentially aid the design of a fully automated treatment planning system that is disease site and patient specific.

## 5 CONCLUSION

A highly efficient patient‐specific collision prevention program for scanning proton therapy has been successfully implemented. The graphical program has provided accurate collision detection since its operation at our institution.

## CONFLICT OF INTEREST

The authors declare that there is no conflict of interest that could be perceived as prejudicing the impartiality of the research reported.

## AUTHOR CONTRIBUTIONS

Jingjing M. Dougherty, Thomas J. Whitaker, Erik J. Tryggestad conceived and planned the studies. Jingjing M. Dougherty, Thomas J. Whitaker, Daniel W. Mundy, Erik J. Tryggestad, and Chris J. Beltran contributed to the interpretation of the results. Jingjing M. Dougherty took the lead in writing the manuscript. All authors provided critical feedback and helped shape the research, analysis, and manuscript.

## Data Availability

The data that support the findings of this study are available from the corresponding author upon reasonable request.
